# RNAcontacts: A Pipeline for Predicting Contacts from RNA Proximity Ligation Assays

**DOI:** 10.32607/actanaturae.11893

**Published:** 2023

**Authors:** S. D. Margasyuk, M. A. Vlasenok, G. Li, Ch. Cao, D. D. Pervouchine

**Affiliations:** Skolkovo Institute of Science and Technology, Moscow, 121205 Russian Federation; College of Life Sciences, Zhejiang University, Hangzhou, Zhejiang, ZJ310058 China; Key Laboratory of RNA Biology, Institute of Biophysics, Chinese Academy of Sciences, Beijing, 100101 China

**Keywords:** RNA, proximity ligation, RNA contacts, splicing, RNA structure

## Abstract

High-throughput RNA proximity ligation assays are molecular methods that are used to simultaneously analyze the spatial proximity of many RNAs in living cells. Their principle is based on cross-linking, fragmentation, and subsequent religation of RNAs, followed by high-throughput sequencing. The generated fragments have two different types of splits, one resulting from pre-mRNA splicing and the other formed by the ligation of spatially close RNA strands. Here, we present RNAcontacts, a universal pipeline for detecting RNA–RNA contacts in high-throughput RNA proximity ligation assays. RNAcontacts circumvents the inherent problem of mapping sequences with two distinct types of splits using a two-pass alignment, in which splice junctions are inferred from a control RNA-seq experiment on the first pass and then provided to the aligner as bona fide introns on the second pass. Compared to previously developed methods, our approach allows for a more sensitive detection of RNA contacts and has a higher specificity with respect to splice junctions that are present in the biological sample. RNAcontacts automatically extracts contacts, clusters their ligation points, computes the read support, and generates tracks for visualizing through the UCSC Genome Browser. The pipeline is implemented in Snakemake, a reproducible and scalable workflow management system for rapid and uniform processing of multiple datasets. RNAcontacts is a generic pipeline for the detection of RNA contacts that can be used with any proximity ligation method as long as one of the interacting partners is RNA. RNAcontacts is available via the GitHub repository https://github.com/smargasyuk/ RNAcontacts/

## INTRODUCTION

The rapid evolution of high-throughput sequencing technology enabled the in vivo identification of spatial contacts between nucleic acids, including the DNA contacts in the 3D chromatin structure [1, 2, 3], functional enhancer-promoter interactions [4, 5], and chromatin-associated RNA–DNA contacts [6, 7]. These methods rest on the basic principle of digestion of nucleic acids cross-linked in macromolecular complexes and subsequent stochastic religation, which occurs predominantly between spatially proximal molecules. Deep sequencing of the resulting chimeric fragments produces hundreds of millions of reads encoding sequence signatures of the interacting loci.

A number of recently developed methods employ RNA proximity ligation to trace back RNA–RNA interactions in vivo and in vitro (see [8] and [9] for review). Some of them, such as PARIS [10], LIGR-seq [11], SPLASH [12], and COMRADES [13], use psoralen derivatives to induce reversible cross-linking between RNA duplexes to assess pairwise structural interactions in vivo with high sensitivity and specificity. In the RIC-seq protocol, the RNA strands are cross-linked through an RNA-binding protein (RBP) [14]. Not only can this approach recapitulate RNA secondary and tertiary structures, but it also helps generate three-dimensional maps of the interacting RNA-RBP complexes. In all these cases, the interactions are encoded within chimeric RNA sequences obtained via digestion and religation.

**Fig. 1:**
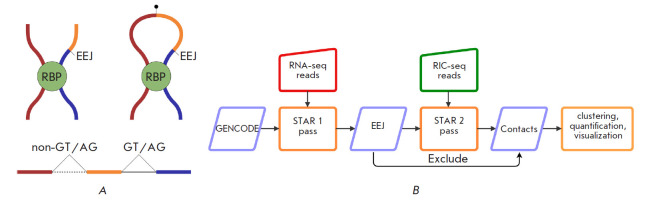
(*A*) – In RIC-seq protocol [14], an unspliced RNA strand can be cross-linked through an RNA-binding protein (RBP) to a strand with exon-exon junctions (EEJ). The sequence formed by proximity ligation aligns to the reference genome with a non-GT/AG split reflecting the ligation point and a canonical GT/AG split resulting from splicing. (*B*) – The RNAcontacts pipeline. On the first pass, short reads from the control RNA-seq experiment are aligned to the reference genome to identify the expressed splice junctions. The latter are used on the second pass as *bona fide *introns when aligning proximity ligation data to detect ligation junctions, which encode RNA–RNA contacts

However, unlike DNA-DNA interactions, which manifest themselves in the sequencing data as split reads that align to a pair of genomic loci, RNA-RNA interactions may produce more sophisticated split alignments because pre-mRNAs are spliced. In particular, the cross-linked fragments may contain both exon-exon junctions and proximity ligation products, thus producing short reads with canonical intronic GT/AG splits resulting from splicing and non-GT/AG splits resulting from religation (Fig. 1A). Accurate mapping of such reads is challenging, because most short-read alignment tools can deal only with one type of split. With just one split model, the aligner would have to make a tradeoff between increasing the penalty for non-GT/AG splits to correctly identify introns at the expense of missing RNA-RNA contacts, or relaxing the GT/AG requirement to correctly detect RNA-RNA contacts, while having incorrect intron mappings. Therefore, developing a computational method for mapping short reads with such distinct types of splits is of particular interest. The current work introduces a computational pipeline that makes it possible to achieve such a goal without developing specialized alignment software.

## EXPERIMENTAL PART

**Genomes and annotations**

The hg19 assembly of the human genome (February 2009) and GENCODE transcript annotation v34lift37 were downloaded from the Genome Reference Consortium [15] and GENCODE website [16], respectively. Intron coordinates were taken from STAR output (see below).

**High-throughput sequencing data**

Two bioreplicates of rRNA-depleted RIC-seq data (GSM3629915 and GSM3629916) in the HeLa cell line [14] were downloaded from the Gene Expression Omnibus under the accession number GSE127188 in FASTQ format. The matched control set of RNA-seq data in the HeLa cell line was downloaded from the ENCODE consortium under the accession numbers ENCLB555ASI and ENCLB555ASJ. On the first pass, the RNA-seq data were mapped to the human genome using the STAR aligner (version 2.7.3a) in paired-end mode with the following additional settings:

--runMode alignReads --outSAMtype BAM SortedByCoordinate --chimOutType Junctions.

On the second pass, the RIC-seq data were mapped to the human genome using the same version of STAR aligner with the following additional settings:

--chimSegmentMin 15 --chimJunctionOverhangMin 15 --chimScoreJunctionNonGTAG -1 --scoreGapNoncan -1 --scoreGapATAC -1 --scoreGapGCAG -1 --chimSegmentReadGapMax 3 --outFilterMatchNminOverLread 0.5 --outFilterScoreMinOverLread 0.5.

The parameter --chimSegmentReadGapMax 3 is introduced to account for the mappability of the additional biotinylated cytosine residue in RIC-seq [14]. The penalty score is reduced to -1 for all non-canonical splice junctions on the second pass.

**Pipeline implementation**

RIC-contacts is implemented in the popular workflow management system Snakemake [17] and is available through the GitHub repository [18]. The input data files are provided through a configuration file in YAML format, which also contains STAR settings and additional parameters that control the minimum distance between two ligation points in a cluster and the cutoff on the distance between neo-junctions to be visualized through UCSC Genome Browser [19]. Neo-junctions were extracted from BAM files using the custom Perl script (neo.pl in RNAcontacts repository) and samtools package v1.14 [20]. The bedops package v2.4.41 was used to cluster ligation points [21]. The number of supporting reads was computed using bedtools package v2.29.0 [22].

**Visualization**

To visualize the contact maps, we converted the contact lists to the ‘cool’ format using the cooler package v0.8.11 with 100-bp resolution and visualized the maps with the pygenometracks package v3.7. Split read visualization was performed with IGV v2.11.2 and the UCSC Genome Browser [19]. By default, only co-linear contacts that span not more than 50,000 nts were visualized through a UCSC Genome Browser track hub (see also the manual [18]).

## RESULTS

**The RNAcontacts pipeline**

We have developed RNAcontacts, a computational pipeline for the analysis of RNA proximity ligation data, which circumvents the problem of multiple split types by aligning short reads in a two-pass mode (Fig. 1B). The workflow is based on the STAR aligner [23]. On the first pass, RNAcontacts aligns the sample-matched set of RNA-seq data in the paired-end mode to identify the splice junctions expressed in a given biological sample using a strict penalty for non-GT/AG splits. Here, RNA-seq represents a control experiment that does not contain fragments resulting from proximity ligation. On the second pass, the reads generated in an RNA proximity ligation experiment are aligned using a relaxed penalty for non-GT/AG splits. At the same time, the splice junctions identified on the first pass as bona fide introns are provided to the input of the second pass, so that the aligner will preferentially make splits at the coordinates from the provided list. Since RNA proximity ligation data may contain chimeric junctions at arbitrary genomic distances or in trans, the alignment on the second pass is performed in the single-end mode. All the split alignments obtained on the second pass are parsed to extract RNA–RNA contacts and exclude the splice junctions obtained on the first pass.

Spliced alignment programs usually generate two separate output files corresponding to co-linear and non-co-linear splits. In particular, the STAR aligner reports co-linear splits (same strand, same chromosome, and forward split orientation) within the standard SAM/BAM output, while non-co-linear splits are placed in the chimeric output file, since the BAM format does not allow their representation with a single CIGAR string [23]. RNAcontacts extracts the coordinates of neo-junctions, i.e., co-linear splits that were found on the second pass from the SAM/BAM output, and combines them with the chimeric splits obtained from the chimeric output. Of note, not only trans, but also cis contacts may be encoded within both neo-junctions and chimeric splits. The combined output of the second pass comprises neo-junctions and chimeric junctions, which are jointly referred to as ligation junctions, with their constituent split positions referred to as ligation points.

In application to the RIC-seq experiment in the HeLa cell line [14], RNAcontacts mapped 94.3% of the ~224 million short reads from the two bioreplicates, with 72.0% of the mapped reads aligned uniquely (see Table S1 for the complete mapping statistics). At the same time, 18.5% of the uniquely mapped reads contained at least one ligation junction, as compared to 3.5%, 2%, and 0.5% previously reported for LIGR-seq, PARIS, and SPLASH, respectively [24]. It is worth noting that spliced alignment programs may differ in their base-wise mapping accuracy and decisions on gap placement [25]. For example, when using the RIC-seq protocol, gap variability can arise even when mapping read mates overlapping the same ligation point because reading the same sequence from one or the other strand may produce slightly different split coordinates due to the lack of consensus sequences that characterize the split (Fig. S1). Furthermore, different copies of the same RNA are digested and religated stochastically, thus resulting in even more considerable variability. Considering this technical and biological variation, we expect to observe clusters of ligation points rather than well-defined junctions, as in GT/AG introns.

Indeed, the distribution of distances between two consecutive ligation points has a rapidly decaying tail, with approximately 50% of distances below 9 nts and 90% of distances below 21 nts (Fig. S2). We chose to cluster the ligation points using single linkage clustering with distance cutoffs (δ) of 10 nts and 20 nts (Fig. S3). A contact was defined as a pair of clusters, with the number of supporting reads equal to the sum of read counts corresponding to ligation junctions.

For each δ, we subdivided the contacts into three groups: intragenic contacts (both ends of a contact belong to an annotated gene), contacts in cis (on the same chromosome, but not in the same gene), and contacts in trans (on different chromosomes). The number of contacts (n), the cluster length (s), the distance between contacting clusters (d, which is defined only for intragenic and cis contacts), and the number of supporting reads (r) were only marginally different for the two values of δ (Table 1). On average, we detected 30% more intragenic contacts than contacts in cis and more than twofold enrichment of contacts in trans with respect to the other two groups. For δ = 10, most clusters had a length of 10 nts (Fig. S4), indicating that they comprise only one individual ligation point surrounded by 5-nt-flanks in both directions.

**Table 1:** The characterization of clusters of RNA–RNA contacts

δ	Metric	Intragenic	in cis	in trans
10	n	1369158	1061470	4881920
	s	10.1±2.2	10.1±2.1	10.2±2.2
log_2_d	10.8±3.3	17.6±4.4	N/A
log_2_r	0.8±0.8	0.5±0.7	0.5±0.7
20	n	1313727	1035656	4851697
	s	20.2±2.4	20.2±2.3	20.3±2.4
log_2_d	11.0±3.3	17.6±4.4	N/A
log_2_r	0.8±0.8	0.5±0.7	0.5±0.7

Note: Clustering distance, δ. The number of contacts, n. Cluster lengths, s. Distance between contacting clusters, d. The number of reads supporting the contact, r. The numbers represent the mean ± standard deviation.

The distances between contacting clusters were distributed differently for neo-junctions and chimeric splits, both in terms of the number of contacts and when weighted by the number of supporting reads (Fig. S5). Remarkably, the distributions have two modes, with the first mode at d-~1000 corresponding to the intragenic contacts encoded by both neo-junctions and chimeric splits. Chimeric reads may encode intragenic contacts if the split is in backward orientation, as in circular RNAs [26]. The second mode for neo-junctions was due to the d ≤ 250 000 condition imposed by the STAR aligner on co-linear splits. However, longer contacts in cis were captured by the second mode of the chimeric distribution. At that, most contacts in cis and in trans were supported by only one read, while most intragenic contacts were supported by two reads (Fig. S6). Therefore, the read support in individual RNA proximity ligation assays is generally quite sparse, even after contacts’ merging into clusters.

**RNAcontacts has higher sensitivity than RICpipe**

**Fig. 2:**
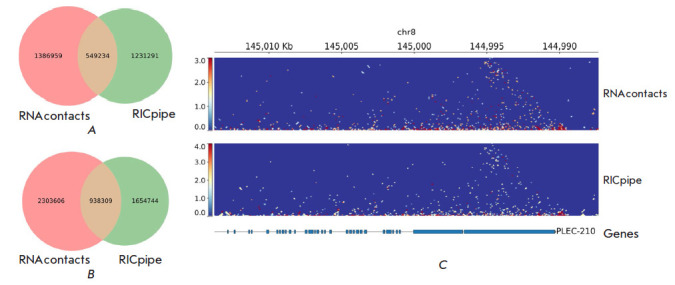
RNAcontacts vs. RICpipe. (*A*) – Venn diagram of ligation junctions obtained by RNAcontacts and RICpipe. (*B*) – Same as A, but weighted by read support. (*C*) – Contact maps for the PLEC-210 gene obtained by RNAcontacts (top) and RICpipe (bottom)

To compare the performance of RNAcontacts with that of RICpipe, a pipeline originally designed to analyze RIC-seq data, we first analyzed ligation junctions of 50 nts or longer located on the same chromosome and then matched their exact genomic positions obtained by the two pipelines. We excluded junctions in reads mapped to rRNA from RNAcontacts results for this analysis, because RICpipe removes rRNA reads [14]. Only 40% of ligation junctions identified by RNAcontacts (compared with 45% by RICpipe) had exactly the same coordinates as the ligation junctions identified by the other pipeline, indicating the differences in the spliced alignment programs (Fig. 2A). However, in terms of the number of short reads supporting the identified ligation junctions, RNAcontacts aligned more reads than RICpipe, indicating an approximately 40% increase in sensitivity (Fig. 2B). When performing the comparison using 100-nts windows, i.e., without exact coordinate matching, we found the results of the two pipelines to be largely concordant. This finding is also evidenced by the similarity of contact maps, with a slightly higher number of contacts for RNAcontacts compared to RIC-pipe (Fig. 2C).

Additionally, we checked the performance of RNAcontacts on the RIC-seq data in the HeLa cell line with and without the first mapping pass. For this purpose, we ran the second pass of RNAcontacts by supplying only the splice junctions annotated in GENCODE [16], without adding the splice junctions inferred for the HeLa cell line on the first pass. As a result, we obtained approximately 1% of spurious ligation junctions corresponding to the endogenous splice junctions in HeLa. We also found that 16,809 out of ~3.5 million ligation junctions identified by RICpipe could be attributed to exon-exon junctions. While the number of such ligation junctions is not large, they are supported by a considerable fraction (> 30%) of reads. Hence, a conclusion is made that the two-pass method provides higher specificity (lower false positive rate) towards RNA–RNA contacts, especially when the transcriptome expressed differs significantly from the annotated one.

## DISCUSSION AND CONCLUSION

In this work, we have presented a conceptual solution to the problem of mapping short reads with two distinct split types, which are characteristic of RNA proximity ligation assays, using the STAR aligner. However, the approach is not limited to STAR, and any other spliced aligner program can be used instead [25]. We have demonstrated that endogenous splice junctions constitute a large portion of the split read alignments in RIC-seq data, and that RNAcontacts allows one to detect split reads aligning to ligation junctions with greater sensitivity than RIC-pipe. The implementation of RNAcontacts in a reproducible and scalable workflow management system Snakemake allows fast and uniform processing of multiple datasets like RIC-seq.

**Fig. 3:**
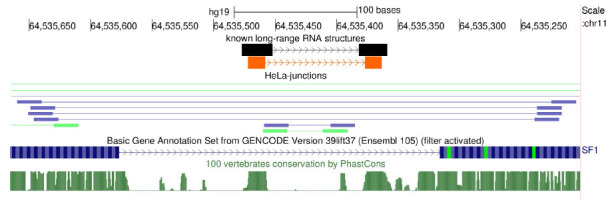
Ligation junctions supporting the RNA structure in the human SF1 gene [28]. The complementary strands are shown in orange. Ligation junctions in the HeLa cell line are shown under the HeLa junctions track. The reads from the two bioreplicates are shown in blue and green

The nature of RNA proximity ligation data is similar to that of Hi-C. Yet it has important distinctions related to the resolution. While it is a common practice for Hi-C to average chromatin contacts at kilobase or megabase scale, the assessment of RNA–RNA contacts using proximity ligation intrinsically targets single-nucleotide levels. Meanwhile, the read support by RIC-seq in the most naturally occurring contacts, for example, mediated by the RNA structure in the human SF1 gene [27], is very weak (see example in Fig. 3). We observed that most RIC-seq contacts in cis and in trans were supported by only one read, raising the issue of assessing the statistical significance of the contacting clusters. This issue should be addressed in future studies supported by larger amounts of data. We expect that many more RNA proximity ligation datasets similar to RIC-seq will soon become available to be analyzed using the RNAcontacts pipeline.

To summarize, RNAcontacts implements a generic RNA–RNA contact analysis pipeline that accounts for multiple split types specific to RNA proximity ligation methods. Initially designed for the RIC-seq protocol, the scope of the software can be extended to any method involving proximity ligation in which one of the interacting partners is RNA.

## AVAILABILITY OF SUPPORTING DATA AND MATERIALS

The data set supporting the results of this study is available in the Zenodo repository (https://zenodo.org/ record/7027475) [27].

## Abbreviations

EEJ: Exon-exon junction
RIC-seq: RNA in situ conformation sequencing
RBP: RNAbinding protein
